# Inhalation of vaccines and antiviral drugs to fight respiratory virus infections: reasons to prioritize the pulmonary route of administration

**DOI:** 10.1128/mbio.01295-23

**Published:** 2023-09-28

**Authors:** Rick Heida, Henderik W. Frijlink, Wouter L. J. Hinrichs

**Affiliations:** 1 Department of Pharmaceutical Technology and Biopharmacy, Groningen Research Institute of Pharmacy, University of Groningen, Groningen, The Netherlands; Icahn School of Medicine at Mount Sinai, New York, New York, USA

**Keywords:** respiratory viruses, mucosal immunity, antiviral agents, inhalation, vaccines, dry powder formulation, pulmonary administration, dry powder inhaler, IgA, aerosols, spray drying, SARS-CoV-2

## Abstract

Many of the current pandemic threats are caused by viruses that infect the respiratory tract. Remarkably though, the majority of vaccines and antiviral drugs are administered via alternative routes. In this perspective, we argue that the pulmonary route of administration deserves more attention in the search for novel therapeutic strategies against respiratory virus infections. Firstly, vaccines administered at the viral portal of entry can induce a broader immune response, employing the mucosal arm of the immune system; secondly, direct administration of antiviral drugs at the target site leads to superior bioavailability, enabling lower dosing and reducing the chance of side effects. We further elaborate on why the pulmonary route may induce a superior effect compared to the intranasal route of administration and provide reasons why dry powder formulations for inhalation have significant advantages over standard liquid formulations.

## PERSPECTIVE

Most of the pandemics of the last centuries have originated from viruses that have evolved to infect the respiratory tract, primarily because, via this route, they can easily be transmitted between hosts (1). For effective vaccination, therefore, it would be intuitive to target the viral portal of entry, thereby exploiting the local immune system that was developed during millennia of evolution. Likewise, it also seems rational to use the pulmonary route of administration for antiviral drugs, since this could multiply the drug’s efficacy at the site of infection. Paradoxically though, among all routes of administration, the pulmonary route is often neglected with the vaccination dogma still being centered around injection-based administration (2) and most of the approved antiviral drugs being administered via the oral route. This is highly remarkable considering the fact that more than 50 years ago, the efficacy of inhaled vaccines in humans was already described (3, 4). Furthermore, a range of bacterial infections and chronic lung diseases are already treated successfully with inhalable drugs for decades (5, 6). We, therefore, argue that, in light of pandemic preparedness, it will be essential not only to focus on the development of novel drugs or vaccines but also to consider the administration route. Specifically, more attention should be paid to the administration of vaccines and antiviral drugs by inhalation in order to directly act at the viral portal of entry, which is the respiratory tract.

## THE RATIONALE BEHIND INHALATION OF VACCINES

While proven successful for measles, mumps, and rubella (MMR) viruses, most respiratory viruses are less effectively counteracted with injected vaccines. This is partly due to the fact that these viruses do not rely on viremia (systemic spread) and have shorter incubation times that are not sufficient for stimulating systemic and long-lasting immune responses. For this reason, the vast majority of the currently licensed injectable vaccines induces only moderate immunity (1). In addition, the high mutation rate of certain viruses (e.g., influenza and SARS-CoV-2) requires the continuous development of novel vaccines that carry (or encode for) epitopes that better match the circulating virus. To this end, the pulmonary route of administration could be of high importance in inducing an immunological response that more closely resembles the response to a natural infection and leads to a broadened immune repertoire.

Unlike injectable vaccines, inhalable vaccines have the capacity to prime local IgA-mediated immune responses that are induced in specialized mucosa-associated lymphoid tissues (MALT) present along the respiratory tract (7). These responses aid in preventing future infections via neutralizing dimeric IgA antibodies that are secreted into the airway lumen (2). The importance of these secretory IgA (SIgA) antibodies in the neutralization of respiratory virus infections has been acknowledged in a plethora of studies, as comprehensively outlined recently by Morens et al. (1). It is therefore of relevance to note that significantly higher levels of IgA antibodies may be reached when the vaccine is targeted to the lungs (8 – 10). Also, IgA has been implicated to be more cross-reactive than IgG (1). Therefore, inhalation of vaccines may lead to an immune response that is more representative of the response following a natural infection. Upon reinfection, this could aid in the neutralization of the virus at the earliest stage, thereby preventing transmission between susceptible individuals. This, in turn, may lead to a reduction in the number of booster doses that are needed for prolonged protective immunity.

The benefits of inhalation as an administration route for vaccines against respiratory viruses (e.g., measles and influenza vaccines) have been confirmed in several studies, as reviewed before (2). Many clinical studies that have assessed the effectiveness of, for instance, inhaled liquid influenza vaccines have shown better protection against reinfection and a reduced occurrence of influenza-related illness compared to patients who received the vaccine via the subcutaneous route. In addition, inhaled forms of measles vaccines have also been shown to lead to superior antibody responses in several cases compared to subcutaneously administered vaccines (2). A few striking examples come from large vaccination trials in school children receiving an aerosolized live-attenuated measles vaccine (11, 12). After revaccination via the aerosol route, seropositive individuals were detected even 6 years after the booster immunization had taken place (12). In contrast, some studies have shown inferiority of the aerosolized method over the injection method, but it is argued that this may be the result of poor delivery of the vaccine to the lower parts of the respiratory tract where the attenuated virus is required to replicate (13). A recent global spike in measles cases, related to a declined vaccination coverage, once again underlines the relevance of alternative immunization strategies that are suitable for mass-vaccination campaigns (14).

Over recent years, there has been an upsurge of studies exploring mucosal routes of administration, especially in light of the COVID-19 pandemic (15). For novel mRNA-based vaccines in particular, it may be beneficial to introduce the vaccine at the natural site of infection (16), considering the fact that Moderna’s novel mRNA-1010 vaccine candidate against influenza has thus far failed to surpass the noninferiority threshold for influenza B strains after parenteral administration (17). Furthermore, the benefits of using the respiratory route for booster vaccination against SARS-CoV-2 have already led to the first-ever conditional approval of a nebulized viral vector vaccine (Ad5-nCoV, produced by CanSino Biologics) for inhalation in China (18, 19). A recently published phase-3 trial has shown that the inhaled vaccine, administered as a heterologous booster, was superiorly effective in inducing seroconversion 14 days post-immunization (reaching a geometric mean neutralizing IgG-titer of 91.4) compared to the intramuscularly injected whole inactivated virus vaccines (19.1). Importantly, it also conferred protection against drifted Omicron variants (20). In addition, the aerosolized vaccine led to superior T cell activation and induced increased levels of both serum- and mucosal IgA antibodies. Furthermore, as reported in a previous trial, using only one-fifth of the dose used for the injection regimen, the vaccine led to less reported side effects than the same vaccine administered intramuscularly (21).

## SHOULDN’T WE GO INTRANASALLY?

When considering the respiratory tract for vaccine delivery, most studies focus on nasal immunization instead of targeting the lungs, as exemplified by SARS-CoV-2 vaccine candidates (15). This is likely due to two reasons. Firstly, nasal immunization is attractive due to its relative ease of administration compared to pulmonary administration. Secondly, there usually is a high abundance of viral attachment receptors on the ciliated epithelia of the upper respiratory tract (22, 23). Although nasal vaccination strategies against SARS-CoV-2 have shown encouraging results in a variety of preclinical studies (24 – 30), studies in humans have thus far failed to meet up their expectations (15, 31, 32), or did not report any clinical trial data (33). A possible explanation that has been given for this discrepancy is that a significant portion of the vaccine may have been swallowed or cleared by the mucociliary escalator before being able to adequately stimulate the immune system (31). In contrast, intranasal instillation of laboratory animals usually happens under mild sedation. Therefore, the swallowing reflex is suppressed, enabling a large proportion of the vaccine to be distributed into the lungs (34). Because of this, there may be a discrepancy between the outcomes of preclinical and clinical studies leading to translational gaps. Also, as the mentioned clinical studies were performed on naive individuals who had no pre-existing memory response against SARS-CoV-2, the immune system may not have been able to rapidly react (31, 32). Supportive of this hypothesis is the fact that the only FDA/EMA-approved intranasal vaccine to date is directed at the influenza virus (FluMist), to which most of the vaccinated population have immunological memory (35, 36). For this reason, we believe the pulmonary route in general could be a more attractive target for generating a robust immune response than the nasal route of administration.

Although not many studies have directly compared the pulmonary route of immunization with the intranasal route (i.e., lower respiratory tract versus upper respiratory tract administration), the pulmonary route has been shown to lead to superior outcomes in both preclinical (13, 37, 38) as well as clinical studies (39). In this regard, some studies have shown that even non-respiratory infections such as human papilloma virus (39) or hepatitis B (40) could benefit from pulmonary administration as long as the vaccine reaches the lower airways. Of interest, also bacterial vaccines have been shown to lead to enhanced protective efficacy against challenges when administered pulmonary compared to intranasally, again favoring the lower respiratory tract as the target site (41, 42). A recent study on a pulmonary administered SARS-CoV-2 subunit vaccine in mice has shown comparable efficacy upon both administration via the pulmonary route and via the intranasal route (43). However, as the mice were anesthetized prior to intranasal instillation (thus enabling the vaccine to distribute into the lungs), it is yet to be determined whether this also holds true for the clinical setting. Nevertheless, a dry powder formulation for inhalation of the vaccine is currently in development (43).

## THE RATIONALE BEHIND INHALATION OF ANTIVIRAL DRUGS

While they are a vital component of global health care, the biggest caveat of vaccines is that they only work prophylactically and need a window of weeks before building an effective and durable immune response. Therefore, antiviral drugs are essential especially during the early stages of a viral outbreak when vaccination campaigns are not yet fully at scale. The importance of this was exemplified at the beginning of the SARS-CoV-2 pandemic during which major hospitalization rates led to an unmanageable burden on hospital care and COVID-19 incidence around the world (44 – 46).

**Fig 1:**
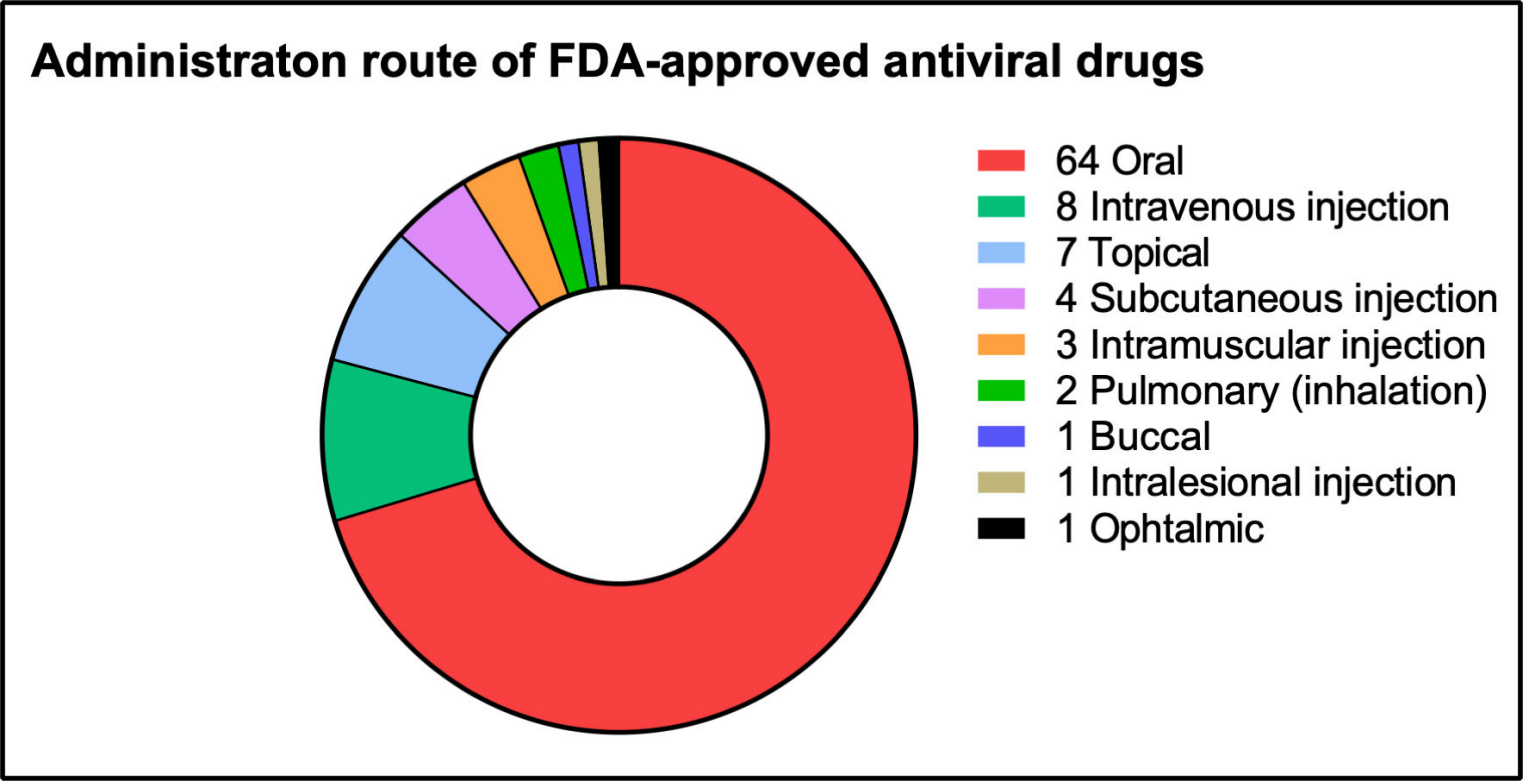
FDA-approved antiviral drugs and combination regimens divided over the administration route (data derived from reference 57 and via a comprehensive search on www.drugs.com). Some drugs that are included in the graph are approved for administration via several routes, namely, acyclovir (oral, intravenous, and buccal), interferon alfa-2b (intramuscular and subcutaneous), letermovir (oral and intravenous), and ribavirin (oral and pulmonary).

As with vaccines, an important aspect of successful treatment is the administration route. In this regard, there are several benefits of using the pulmonary route for the administration of antiviral drugs against respiratory viruses. In contrast to orally administered drugs, direct administration to the respiratory tract causes superior bioavailability of the drug at the primary site of action as it does not rely on the systemic circulation to be delivered at the target site and is, therefore, not affected by the hepatic first-pass effect (47). This enables lower dosing. As a consequence, the chances for (severe) adverse events are lower. Especially for respiratory viruses such as SARS-CoV-2 and influenza, inhaled antivirals can be a good addition to the currently approved treatment regimens, as has been pointed out in several studies (48 – 53). Considering these benefits, it is highly remarkable that, in addition to vaccines, the pulmonary route of administration is not used more often for the administration of antiviral drugs. While three out of 10 treatable viruses infect the respiratory tract, only two out of the ±80 currently FDA-approved antiviral drugs and combination therapies (2.5%) can be administered via inhalation (i.e., ribavirin and zanamivir). In fact, the most common administration route for antiviral drugs is still the oral route (Fig. 1). This is remarkable as inhalation of pharmaceutical compounds for the treatment of common lung diseases is widespread (5) and has its origin in ancient history (54). Also, bacterial infections of the lower respiratory tract are already treated successfully with inhalable antibiotics and several other inhaled antibiotics are currently in development (55). Furthermore, even pulmonary bacteriophage therapy is gaining traction (56).

## WHY DRY FORMULATIONS OF VACCINES AND ANTIVIRAL DRUGS FOR INHALATION ARE THE WAY TO GO

Although mRNA vaccines against COVID-19 have proven to be highly efficacious in preventing severe disease, they require storage (and transportation) at −80º or −20º which puts an enormous burden on the distribution of vaccines around the world. We, therefore, argue that one of the major opportunities for the field of pulmonary administered vaccines and antiviral drugs is to formulate them as thermostable dry powders. The advantage of this is that, when formulated correctly either with or without the use of stabilizing excipients, they can be stored at ambient temperatures because they are less thermosensitive than liquid formulations. In addition, they are lighter in weight which benefits bulk storage and transportation. Another advantage of dry powder formulations compared to liquid formulations is that they can be administered via single-use inhalers that are easier to operate by the patient and can be disposed of (or even recycled) afterwards (58). Such dry powder inhalers do not rely on laborious procedures that require an external power source, as is the case with for instance nebulized ribavirin treatment against respiratory syncytial virus infection. This may tremendously increase both patient compliance and comfort. Also, in contrast to metered-dose inhalers, dry powder inhalers can carry drugs that need to be administered at a high dose. Apart from these advantages, to our best knowledge, no single inhalable dry powder vaccine is currently in (clinical) development. The only dry powder formulation that is currently approved by the FDA for inhalation is the anti-influenza drug zanamivir.

With regard to dry powder vaccine formulations for inhalation, it needs to be ensured that a proper drying technique is used that (i) can be scaled up for industrial applications, (ii) yields particles with a proper (aerodynamic) size distribution, and (iii) is not detrimental to the chemical integrity of the vaccine. A method that has been widely explored in this regard is spray drying, as comprehensively reviewed recently (59). With this technique, the vaccine solution is dried under a hot stream of inert gas in a tweakable process, yielding particles with a desirable size distribution. By incorporating stabilizing glass-forming excipients, such as sugars like inulin or trehalose, the vaccine can be protected from heat-, shear-, and evaporation-induced stresses. Using the spray drying method, various types of vaccines have been successfully processed into dry powders for inhalation while retaining their activity, such as whole-inactivated virus vaccines, live-attenuated vaccines, and subunit vaccine formulations (59, 60).

Arguments against the use of dry powder formulations for inhalation have recently been outlined (61). They include that the effectiveness of an inhaled drug or vaccine may be limited by the capacity of the patient to perform a correct inhalation maneuver. If performed incorrectly, this may lead to differences in the delivered dose. It is, therefore, of importance that inhalers are designed in such a way that both makes the inhaler’s performance insensitive to the naturally occurring variations in inhalation and that ensures that a correct inhalation is performed upon intuitive use. In addition, the patient should receive clear instructions about the correct inhalation technique and the necessity of therapy adherence (61). Other arguments involve the potential environmental impact of dry powder inhalers. However, partly due to their suitability for recycling, this does not necessarily outweigh the impact of syringe- and needle waste. Moreover, as the highly energy consuming cold chain can be omitted for these thermostable and relatively lightweight vaccine and antiviral drug powders, the environmental impact as well as potentially increased costs are in part canceled out. Nevertheless, in relation to the inability of some patient groups (e.g., infants and cognitively or physically impaired patients) to correctly use inhalers, the importance of offering multiple administration routes, i.e., oral or injection-based or via nebulization methods remains.

## A CALL FOR ACTION

Although the emergence of SARS-CoV-2 in 2019 initiated an unmet acceleration in research and development of both vaccines and antiviral drugs, the pressure of (emerging) viruses with pandemic potential has not yet declined. In fact, while we are still in the aftermath of the COVID-19 crisis, the next pandemic might already be lurking around the corner. An example of such a pandemic threat is the H5N1 bird flu, which has killed an astounding number of (at least) 50 million birds in recent years and has already caused multiple spillovers to mammals, including humans (62). Largely due to external factors such as (the indirect effects of) climate change (63, 64), intensive farming (65, 66), an increasing world population, globalization, and its effect on international travel (67), emerging (zoonotic) viruses can spread rapidly, employing an efficient infrastructure that is not limited by borders (68). One of the major lessons that should be learned from COVID-19 and previous pandemics is that pandemic preparedness is of major importance for effective containment. While protective measures such as lockdowns and face masks have been essential in putting the break on the viral spread, parallel development of potent antiviral drugs and novel vaccines has been proven of utmost importance. In light of this, we believe that the administration route is key to achieving the best therapeutic outcome. Specifically, the pulmonary route of administration should no longer be neglected.
